# Self-reported history of Pap-smear in HIV-positive women in Northern Italy: a cross-sectional study

**DOI:** 10.1186/1471-2407-10-310

**Published:** 2010-06-21

**Authors:** Luigino Dal Maso, Silvia Franceschi, Mauro Lise, Priscilla Sassoli de' Bianchi, Jerry Polesel, Florio Ghinelli, Fabio Falcini, Alba C Finarelli

**Affiliations:** 1 Epidemiology and Biostatistics Unit, Centro di Riferimento Oncologico (CRO) - IRCCS, Via Gallini 2, 33081 Aviano (PN), Italy; 2 Dipartimento di Medicina del Lavoro "Clinica del Lavoro Luigi Devoto," Sezione di Statistica Medica e Biometria "GA Maccacaro," Università degli Studi di Milano, Milan, Italy; 3 International Agency for Research on Cancer, cours Albert Thomas 150, 69372 Lyon cedex 08, France; 4 Servizio Sanità Pubblica, Assessorato Politiche per la Salute - Regione Emilia-Romagna, Viale Aldo Moro 21, 40127 Bologna, Italy; 5 Presidente commissione consultiva tecnico-scientifica per la promozione di interventi per la prevenzione e la lotta contro l'AIDS, Regione Emilia-Romagna, Viale Aldo Moro 21, 40127 Bologna, Italy; 6 Romagna Cancer Registry, Department of Medical Oncology, Cancer Institute of Romagna (IRST), Via Piero Maroncelli 34/36, 47014 Meldola, Italy

## Abstract

**Background:**

The incidence of invasive cervical cancer in HIV-positive women is higher than in the general population. There is evidence that HIV-positive women do not participate sufficiently in cervical cancer screening in Italy, where cervical cancer is more than 10-fold higher in women with AIDS than in the general population. The aim of the present study was to evaluate the history of Pap-smear in HIV-positive women in Italy in recent years. We also examined the sociodemographic, clinical, and organizational factors associated with adherence to cervical cancer screening.

**Methods:**

A cross-sectional study was conducted between July 2006 and June 2007 in Emilia-Romagna region (Northern Italy). All HIV-positive women who received a follow-up visit in one of the 10 regional infectivology units were invited to participate. History of Pap-smear, including abnormal smears and subsequent treatment, was investigated through a self-administered anonymous questionnaire. The association between lack of Pap-smear in the year preceding the interview and selected characteristics was assessed by means of odds ratios (OR) and 95% confidence intervals adjusted for study centre and age.

**Results:**

A total of 1,002 HIV-positive women were interviewed. Nine percent reported no history of Pap-smear, and 39% had no Pap-smear in the year prior to the date of questionnaire (last year). The lack of Pap-smear in the last year was significantly associated with age <35 years (OR = 1.4, compared to age ≥45 years), lower education level (OR = 1.3), first HIV-positive test in the last 2 years (OR = 1.4), and CD4 count <200 cells/μl (OR = 1.6). Conversely, when women were advised by a gynecologist rather than other health workers to undergo screening, it significantly increased adherence. Non-significantly higher proportions of lack of Pap-smear in the last year were found in women born in Central-Eastern Europe (OR = 1.8) and Africa (OR = 1.3). No difference in history of Pap-smear emerged by mode of HIV-acquisition or AIDS status.

Three hundred five (34%) women reported a previous abnormal Pap-smear, and of the 178 (58%) referred for treatment, 97% complied.

**Conclusions:**

In recent years the self-reported history of Pap-smear in HIV-positive women, in some public clinics in Italy, is higher than previously reported, but further efforts are required to make sure cervical cancer screening is accessible to all HIV-positive women.

## Background

The incidence of invasive cervical cancer in HIV-positive women is higher than in the general population [1]. This elevated incidence varies from country to country depending on study site and characteristics of the populations under study [2-4]. The weaker association between HIV-infection and cervical cancer risk in some countries seems to be explained by competing risks of death (e.g.: in Africa), or early detection of pre-invasive lesions (e.g.: in the United States) [5].

Effective screening and early treatment of precancerous cervical lesions are key factors in preventing the progression to invasive cervical cancer in both HIV-positive and -negative women [6]. Recent guidelines recommend that, following two initial normal Pap-smears at a 6-month interval, all HIV-positive women should undergo annual cervical cytologic examination [7]. In addition, it is recommended that all immunosuppressed women with atypical squamous cells undergo colposcopy [7].

In the United States, approximately 80% of HIV-positive women reported a history of Pap-smear in the past year [8,9]. Low incidence rates of invasive cervical cancer, similar to those among HIV-negative women, were found among adequately screened HIV-positive women [10]. In contrast, there is evidence that HIV-positive women do not participate sufficiently in cervical cancer screening in Southern European countries such as Italy and Spain, where cervical cancer incidence is more than 10-fold higher in women with AIDS than in general population [3,5,11,12].

In Italy, one-half of women with invasive cervical cancer as the AIDS-defining illness (3% of all women with AIDS) had their first HIV-positive test ≥10 years before cancer diagnosis [12]. This long interval suggests a failure to stop the progression of precancerous lesions through screening, despite the knowledge of HIV infection.

A large cross-sectional study showed not only a scanty use of Pap-smear among HIV-positive Italian women, but also that these women did not know that a Pap-test was used to prevent cervical cancer [13]. In addition, a survey conducted among clinicians in 27 Italian HIV centers showed low compliance with the published guidelines on gynecologic follow-up of HIV-positive women in Italy [14].

The aim of the present study was to evaluate the recent history of Pap-smear in HIV-positive women in Italy followed in public HIV clinics. We also examined the sociodemographic, clinical, and organizational factors associated with the lack of adherence to screening recommendations.

## Methods

### Study population

A cross-sectional study was conducted between July 2006 and June 2007 the in Emilia-Romagna Region (Northern Italy). At end of 2006, this region covered an area of more than 22.000 km^2 ^(7% of Italy), with a population of 4.2 million inhabitants (7% of Italian population). Approximately 1,500 HIV-positive women are estimated to live in the Emilia-Romagna Region [15,16]. All HIV-positive women who underwent a follow-up visit (recommended every 6-months) in one of the 10 regional infectivology units were invited to participate. The study period was limited to one year in order to reduce the possibility of duplication.

A total of 1,108 HIV-positive women were invited and 1,002 (90.4%) accepted to participate. Study aims were explained and written informed consent was obtained from study women, who subsequently completed a self-administered anonymous questionnaire. History of Pap-smear was investigated in detail to assess lifetime screening, including dates of first and last Pap-smear, and number of Pap-smears in the three years preceding the questionnaire. The questionnaire included additional items on the place where Pap-smear was taken, HPV testing, history of abnormal smears, and treatment of cervical lesions. Other collected data included sociodemographic factors (e.g., age, education level, and country of birth), smoking and reproductive history, sexual habits (e.g., age at first intercourse, lifetime number of sexual partners, and use of contraceptive methods), and history of HIV infection (e.g., date of first HIV-positive test and most probable route of HIV acquisition). In a separate form, the attending infectivologist reported information on the course of HIV-infection (e.g.: HIV-related hospitalization, last CD4 count and HIV RNA values, AIDS status, and AIDS-defining conditions).

The Ethical Committees of all participating centers approved the study protocol.

### Statistical analysis

Odds ratios (ORs) and 95% confidence intervals (CIs) were used as the measures of association between history of Pap-smear and women's characteristics, and were estimated using logistic regression models adjusted for study centre and age [17,18]. Estimates were also calculated including further adjustment for area of birth, mode of HIV acquisition, and time since first HIV-positive test.

## Results

A total of 1,002 HIV-positive women completed the questionnaire. The median age was 41 years and the vast majority of women (87%) fell in the 30-to-49-year age range. Eighty percent of our study women were born in Italy and nearly two-thirds reported sexual intercourse as the route of HIV acquisition. One hundred eighty (18%) women had a previous AIDS diagnosis and 56% had their first HIV-positive test 10 or more years prior to the questionnaire.

**Figure 1:**
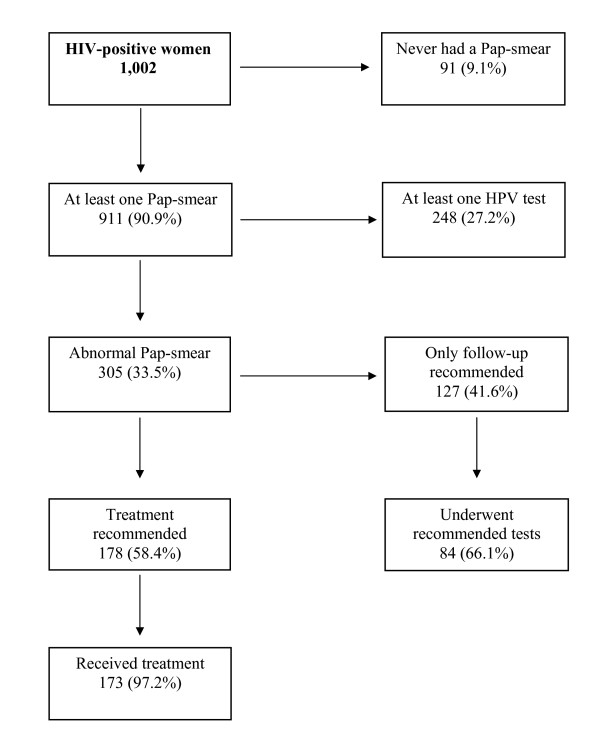
**Pap-smear practice and treatment after a positive Pap-smear in HIV-positive women**. Emilia-Romagna Region, Italy, 2006-2007

Nine percent of women reported no history of Pap-smear and 305 (34%) had abnormal Pap-smear results (Figure 1). Women with no history of Pap-smear were younger and more likely to be born in Central-Eastern Europe (15%) or Africa (29%) than women reporting previous Pap-smears (3% and 9%, respectively) (data not shown). HPV test was reported by only 27% of women (Figure 1).

Overall, 607 (61%) of the 1,002 HIV-positive women had a Pap-smear in the year prior to questionnaire (last year, Table 1). Women younger than 35 years (OR, compared to age ≥45 = 1.4; 95%CI: 1.0-2.0), those with lower education level (OR = 1.3; 95%CI: 1.0-1.7), those with recent first HIV-positive test (OR for ≤2 years vs ≥10 years = 1.4; 95%CI: 1.0-2.1), or a CD4 count of <200 cells/μl (OR compared to ≥500 = 1.6; 95%CI: 1.0-2.5) were more likely to have no history of Pap-smear in the year before questionnaire. Non-significantly higher proportions of lack of Pap-smear in the last year were found in women born in Central-Eastern Europe (OR = 1.8; 95%CI: 0.9-3.6) and Africa (OR = 1.3; 95%CI: 0.8-2.0). No difference in Pap-smear history emerged by mode of HIV acquisition, or AIDS status.

**Table 1:** Odds ratio (OR) and corresponding 95% confidence intervals (CI) for lack of Pap-smear in the last year by selected characteristics

	Pap-smear in the last year							
	**Yes**		**No**		**OR**^**†**^	**(95%CI)**	**OR**^‡^	**(95%CI)**
	
	**n**	**(%)**	**n**	**(%)**				

**Total**	607	(60.6)	395	(39.4)				
								
**Age at interview (years)**								
≥45	145	(58.7)	102	(41.3)	1		1	
40-44	227	(65.6)	119	(34.4)	0.8	(0.5-1.1)	0.8	(0.5-1.1)
35-39	131	(63.3)	76	(36.7)	0.8	(0.6-1.2)	0.8	(0.6-1.2)
<35	104	(51.5)	98	(48.5)	1.4	(1.0-2.0)	1.2	(0.8-1.8)
**Education level**								
High school/University	255	(63.1)	149	(36.9)	1		1	
Middle school/Primary	241	(55.9)	190	(44.1)	1.3	(1.0-1.7)	1.3	(1.0-1.7)
**Area of birth**								
Italy	495	(62.1)	302	(37.9)	1		1	
Abroad	112	(54.6)	93	(45.4)	1.2	(0.8-1.7)	1.1	(0.8-1.6)
Central-Eastern Europe^**§**^	17	(42.5)	23	(57.5)	1.8	(0.9-3.6)	1.7	(0.8-3.3)
Africa	56	(53.3)	49	(46.7)	1.3	(0.8-2.0)	1.1	(0.7-1.9)
Other countries	39	(65.0)	21	(35.0)	0.8	(0.5-1.5)	0.9	(0.5-1.5)
**Mode of HIV acquisition**								
Sexual	336	(62.1)	205	(37.9)	1		1	
Injecting drug use	164	(61.0)	105	(39.0)	1.2	(0.9-1.6)	1.1	(0.8-1.6)
Other/Unknown	107	(55.7)	85	(44.3)	1.2	(0.8-1.6)	1.1	(0.7-1.5)
**Time since first HIV+ test (years)**								
≥10	357	(63.2)	208	(36.8)	1		1	
3-9	169	(62.1)	103	(37.9)	0.9	(0.7-1.3)	0.9	(0.7-1.3)
≤2	78	(48.4)	83	(51.6)	1.4	(1.0-2.1)	1.3	(0.9-2.0)
**Previous AIDS diagnosis**								
No	504	(61.3)	318	(38.7)	1		1	
Yes	103	(57.2)	77	(42.8)	1.2	(0.9-1.7)	1.2	(0.9-1.7)
**CD4 count at last visit (cells/μl)**								
≥500	284	(63.5)	163	(36.5)	1		1	
200-499	271	(58.8)	190	(41.2)	1.2	(0.9-1.6)	1.2	(0.9-1.5)
<200	45	(51.7)	42	(48.3)	1.6	(1.0-2.5)	1.6	(1.0-2.6)
**Screening advice received from**								
Infectivologist	244	(66.8)	121	(33.2)	1		1	
Gynecologist	122	(77.2)	36	(22.8)	0.6	(0.4-0.9)	0.6	(0.4-1.0)
Screening program	61	(59.8)	41	(40.2)	1.4	(0.9-2.2)	1.3	(0.8-2.1)
Do not remember	180	(47.7)	197	(52.3)	2.1	(1.5-2.8)	2.2	(1.6-2.9)
**Hospitalized for HIV**								
No	382	(61.8)	236	(38.2)	1		1	
Yes	225	(58.6)	159	(41.4)	1.2	(0.9-1.5)	1.2	(0.9-1.5)
**Abnormal Pap-smear**^**#**^								
Never	366	(61.7)	227	(38.3)	1		1	
Ever	235	(77.0)	70	(23.0)	0.5	(0.3-0.6)	0.4	(0.3-0.6)

Emilia-Romagna region, Italy, 2006-2007*

* Figures may not add up to totals because of missing values. ^†^Estimates from the logistic regression models adjusted for age and study centre; ^‡^Adjusted as ^† ^plus terms for area of birth, mode of HIV acquisition, and time since first HIV-positive test. ^**§**^Included Albania, Bulgaria, Former-Yugoslavia, Poland, Romania, Hungary, Former-USSR, Czech Republic and Slovakia, Cyprus; ^**#**^Women reporting no previous Pap-smear were excluded

Receiving screening advice from a gynecologist rather than an infectivologist (OR = 0.6; 95%CI: 0.4-0.9) and history of abnormal Pap-smear (OR = 0.5; 95%CI: 0.3-0.6) were associated with better screening participation (Table 1). The multivariate models, including additional terms for area of birth, mode of HIV acquisition, and time since first HIV-positive test, did not materially modify the risk estimates even if associations with age and first HIV-positive test were not statistically significant (Table 1).

Among the 305 women who reported a previous abnormal Pap-smear, treatment was recommended for 178 (58%); of whom, 173 (97%) complied. Among the 127 (42%) women who were referred for further testing but not treatment, 84 (66%) underwent all the recommended follow-up tests.

Among the 178 women to whom treatment was recommended, 145 (81%) reported colposcopy and all follow-up tests. Conization was the surgical procedure most frequently reported by patients (122, 69%), followed by hysterectomy (19, 11%), and cryotherapy (8, 4%).

## Discussion

This study was the first attempt to provide estimates of self-reported Pap-smear history in HIV-positive women attending infectivology units of a large Italian region. We found that 91% of women had had at least one Pap-smear in their lifetime, and 61% reported a Pap-smear in the last year. These proportions are similar to the ones reported by the general population of the same age and geographical area (95% and 47%, respectively) (Carrozzi and Bertozzi, personal communication), and substantially higher than those reported (43%) in 2001 by a physician-based Italian survey [14]. Increase in the number of Pap-smears performed, however, is difficult to evaluate, given the different study designs of previous studies conducted in Italy [13,14]. The proportion of women who had a Pap-smear in the last year, however, remains suboptimal with respect to current guidelines regarding HIV-positive women (annual Pap-smear recommended to all patients), and is lower than the estimates (approximately 80%) reported in the USA [8,9].

Factors associated with lack of recent Pap-smear were young age (<35 years), recent HIV diagnosis (≤2 years), and more advanced diseases (CD4 count at last visit <200 cells/μL). Conversely, receiving screening advice from a gynecologist significantly improved the adherence to Pap-smear. We found less influence of education level or country of birth than a previous cross-sectional study conducted in Rome [13]. As expected, study women reported a high proportion of previous abnormal Pap-smears (34%), in agreement with previous reports among HIV-positive women in Italy [13] and the United States [8,9].

Overall, our study highlights the difficulties in following the recommended protocol of combining routine follow-up of HIV infection with gynecological examination, which is generally not performed in infectivology units [5,10]. In addition, patient compliance needs to be improved [14] and health professionals (e.g., infectious disease specialists and gynecologists) should thoroughly inform HIV-positive women (particularly the disadvantaged ones) of the importance of Pap-smear to their health.

The present study has strengths and weaknesses. Strengths included the high participation among unselected women attending infectivology units. Approximately 1,500 HIV-positive women are estimated to live in the Emilia-Romagna Region [15,16] and all women attending HIV follow-up (recommended every 6 months) were contacted in a 1-year period. A previous study in Northern Italy [19] showed that patients infected with HIV through injecting drug use, patients without AIDS diagnosis, or patients with higher CD4 counts are more likely to miss medical appointments and discontinue their follow-up. It is unlikely that HIV-positive women in the region received their follow-up visits elsewhere, given the high standard of medical care provided (free of charge) in Emilia-Romagna in comparison with other Italian areas. However, the possibility of some socio-demographic bias in women who did not attend regularly follow-up cannot be totally ruled out.

The number of self-reported Pap-smears among HIV-positive women in our study may be an overestimate as women tend to over-report their participation in cervical cancer screening in a given timeframe [20]. Confidentiality prevented us from linking women's reports with gynecological and cytological records, thus leaving substantial uncertainty about the actual additional tests and treatments performed. To validate self-reported Pap-smear use and to follow-up the treatment of HIV-positive women diagnosed with gynaecological lesions, a new study is being planned, which will adopt broader confidentiality rules and obtain a written consent to follow-up. The most important limitation of our survey, however, is the restriction to HIV-positive women followed by public clinics in one of the best organized regions of Italy, in terms of participation and quality of cervical cancer screening [21,22].

## Conclusions

Our study showed that progresses have been made to screen adequately HIV-positive women in Italy and that such efforts can also reach the most vulnerable populations (e.g.: immigrants). The opportunity to prevent cervical cancer in women living with HIV infection should not be missed; successful implementation of such screening programs will also teach valuable lessons that could then be applied to all women [5]. There is, however, still some scope for improvement, especially in the completeness of treatment and follow-up of HIV-positive women with an abnormal Pap-smear. Most importantly, much remains to be done to make sure that the experience of the Emilia-Romagna Region can be extended to other parts of Italy.

## Competing interests

The authors declare that they have no competing interests.

## Authors' contributions

LDM, SF, PSdB, and ACF conceived the study and were involved in data interpretation. PSdB coordinated the data collection. ML and JP collaborated with LDM in the acquisition of data and statistical analyses. LDM drafted the manuscript. FG and FF supervised the study. All members of the "Screening of HIV-positive women in Emilia-Romagna (SHER) Study" actively collaborated to all the phases of the study. All Authors critically revised the manuscript for important intellectual contents. All authors read and approved the final manuscript.

## Authors' information

LDM is a Senior Scientist at Aviano Cancer Institute and coordinated a surveillance study on cancer in people with HIV/AIDS in Italy. SF is Head of Infections and Cancer Epidemiology Group at International Agency for Research on Cancer (Lyon, France) and has been carrying out numerous international studies on the association between infection and cancer. ACF is Head of Public Health department of Emilia-Romagna Region. ML and JP collaborated with LDM, PSdB collaborated with ACF. FG is president of AIDS Commission, Emilia-Romagna Region, FF is Chief of Romagna Cancer Registry.

## Pre-publication history

The pre-publication history for this paper can be accessed here:

http://www.biomedcentral.com/1471-2407/10/310/prepub
